# Leber’s hereditary optic neuropathy: course of disease in consideration of idebenone treatment and type of mutation

**DOI:** 10.1007/s00417-020-05045-4

**Published:** 2020-12-18

**Authors:** Felix Tonagel, Helmut Wilhelm, Paul Richter, Carina Kelbsch

**Affiliations:** Centre for Ophthalmology, University of Tuebingen, Elfriede-Aulhorn-Str. 7, 72076 Tuebingen, Germany

**Keywords:** Leber’s hereditary optic neuropathy, LHON, Recovery, Improvement, 11778, Idebenone

## Abstract

**Purpose:**

In September 2015, the first and so far only medication for treatment of Leber’s hereditary optic neuropathy (LHON) was approved in the EU. The drug in question is idebenone (©Raxone) and has been given to all newly diagnosed patients of the University Eye Hospital Tuebingen since the approval of the drug. The aim of the study was to find out whether regular administration of the drug led to an improvement in vision. We retrospectively examined 2 cohorts of consecutive patients with newly occurred visual impairment and LHON diagnosis: One with the initial diagnosis made from January 2010 until April 2014 and a second from October 2015 until January 2020.

**Methods:**

Retrospective, observational cohort study. All electronic medical files of newly diagnosed and genetically confirmed LHON patients of the University Eye Hospital Tuebingen from January 2010 until April 2014 (cohort 1) and October 2015 until January 2020 (cohort 2) with at least 12 months of follow-up examinations have been analyzed.

**Results:**

Five patients were included in the first and 7 patients in the second cohort. Patients of cohort 1 received no medication; patients of cohort 2, a daily dose of 900 mg idebenone. The primary visual acuity (VA) ranged between 0.03 and 0.5 in cohort 1 and did not improve during the observation period (median 60 months, range 23–87 months). The patients of cohort 2 have been observed for a median of 23 months (range 12–35 m). The primary VA ranged from 0.01 to 0.16. A recovery in one or both eyes with a final VA from 0.8 to 1.0 was experienced in 3 out of 7 patients. All patients showing a recovery of VA carried the m.11778G>A mutation.

**Conclusion:**

The observed improvement in the treated cohort may be considered as a hint on the efficacy of idebenone in LHON. The time course of improvement suggests that idebenone should be given 1.5 years in newly diagnosed LHON cases.

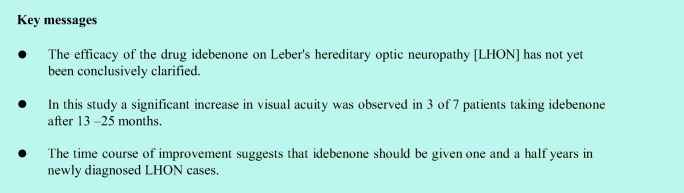

## Introduction

Leber’s hereditary optic neuropathy (LHON) is one of the most common mitochondrial diseases. The reported prevalence fluctuates between 1:45,000 [1] and 1:31,000 and in males 1:14,054 [2]. Through damage to retinal ganglion cells, a severe loss of visual acuity (VA) is incurred accompanied by central scotoma.

The probability of spontaneous improvement depends on the underlying mutation type: m.14484T>C has the best prognosis of improvement in 37–71%, whereas m.11778G>A and m.3460G>A mutations have only a 4% chance in the USA and Europe [3, 4]. Japanese m.11779G>A patients seem to have a better prognosis of up to 17% [5]. In September 2015, the first and so far only medication for treatment of LHON was approved in the EU. The drug in question is idebenone (©Raxone), a short-chain benzoquinone [2,3-dimethoxy-5-methyl-6-(10hydroxydecyl)-1,4-benzoquinone] that supports the mitochondrial electron transport chain [6]. Idebenone has been given to all newly diagnosed patients of the University Eye Hospital Tuebingen since the approval of the drug. The aim of the study was to find out whether regular administration of the drug led to an improvement in vision. We retrospectively examined 2 cohorts of patients with newly occurred visual impairment and LHON diagnosis: one with the initial diagnosis made from January 2010 until April 2014 (no treatment) and a second from October 2015 until January 2020 (idebenone 900 mg daily).

## Materials and methods

This study was approved by the local ethics committee of the faculty of medicine of the Eberhardt-Karls-University Tuebingen. The study was planned as a retrospective, observational cohort study and within the neuro-ophthalmology unit of the University Eye Hospital in Tuebingen that is a tertiary care ophthalmic clinic.

All electronic medical files of newly diagnosed and genetically confirmed LHON patients of the University Eye Hospital Tuebingen from January 2010 until April 2014 (cohort 1) and October 2015 until January 2020 (cohort 2) with at least 12 months of follow-up examinations have been analyzed. Eyes with other causes of visual loss than LHON were excluded from further data analysis. In the first cohort, no medication was given (as not available at that time); in the second cohort, all LHON patients received idebenone 900 mg daily. VA values are represented in decimal notation. According to Lange et al. [7], we have converted the VA “counting fingers” to 0.010, “hand motion” to 0.0052, and “light perception” to 0.

All statistical analyses were performed using JMP® 14.2.0 statistical software (SAS Institute, Cary, NC, USA) and SPSS® statistical software (IBM, Armonk, NY, USA).

## Results

Ten patients of the first and 6 patients of the second cohort were excluded due to an insufficient follow-up period. Of these, 11 carried the m.11778G>A, 2 the m.3635G>A, and one each the m.3890G>A, m.3460G>A, and m.9438G>A mutation. Five patients were included in the first and seven patients in the second cohort. M.14484T>C mutation was not detected during the observation period.

Patient number 7 of the second cohort had a history of severe visual loss of his left eye caused by retinal detachment. This eye was consequently excluded from further data analysis. There was one female patient in each cohort in total (Tables 1 and 2). Median age in cohort 1 was 23 years (range 12–48 years) and 28 years (range 14–48 years) in cohort 2.

**Table 1:** Cohort 1 (no treatment): Patient characteristics and course of visual acuity

No.	Age	Sex	Mutation	Homo-/heteroplasmy	Initial visual acuity		Deterioration (time since onset)	Final visual acuity		Observation period (m)
					OD	OS		OD	OS	
1	30	M	11778	Homoplasmy	0.10	0.05		0.05	0.05	87.00
2	26	M	3460	N/A	0.03	0.50		0.03	0.50	60.00
3	48	F	3460	Heteroplasmy	0.10	0.16		0.01	0.01	23.00
4	17	M	11778	Homoplasmy	1.00	0.05	0.3 (OD, 3 m)	0.04	0.04	72.00
5	12	M	11778	N/A	0.30	0.50		0.03	0.03	24.00

*m* months

**Table 2:** Cohort 2 (idebenone 900 mg daily): Patient characteristics and course of visual acuity

No.	Age	Sex	Mutation	Homo-/heteroplasmy	Initial visual acuity		Deterioration (time since onset)	Final visual acuity		Observation period (m)	Idebenone recovery (m)
					OD	OS		OD	OS		
6	18	M	11778	Homoplasmy	0.03	*0.63*	*0.05 (OS, 2 m)*	0.05	*0.80*	25	13.00
7	33	M	11778	Homoplasmy	*0.03*	*		*1.00*	*	32	25.00
8	28	M	11778	Homoplasmy	*0.16*	*0.10*		*0.80*	*0.80*	20	15.00
9	14	M	MT-ND1-3733	Homoplasmy	0.03	0.01		0.0052	0.02	12	
10	15	M	11778	Homoplasmy	0.02	0.03		0.0052	0.05	23	
11	34	F	MT-ND1-3635	Heteroplasmy	0.10	0.50		0.01	0.50	35	
12	48	M	3460	Heteroplasmy	0.63	0.00	0.04 (OD, 1 m)	0.01	0.00	20	

Eyes that have improved are highlighted in italics

*m* months

*Patient 7: pre-existing blindness after retinal detachment of left eye

### Cohort 1

In cohort 1, three patients carried the m.11778G>A and 2 patients the m.3460G>A mutation. Primary VA differed between 0.03 and 1.0 (median 0.1) in the right eye and 0.05 and 0.5 in the left (median 0.16). One patient suffered a deterioration of his better eye (VA 1.0 to 0.3) 3 months after onset of first symptoms. VA did not improve during the observation period (median 60 months, range 23–87 months): Final VA was 0.01–0.05 for the right eye (median 0.03), and 0.01–0.5 (median 0.04) for the left eye. Information on homo-/heteroplasmy of the mutation was available in 3 of the 5 patients: 2 patients showed homoplasmy, one heteroplasmy.

Table 3 shows the global and temporal retinal nerve fiber layer (RNFL) thicknesses of all patients. The median global RNFL of cohort 1 was 46 μm (39–130); the temporal RNFL 27 μm (23–113).

**Table 3:** Global and temporal RNFL thicknesses

No.	OD		OS	
	global	Temp	global	Temp
1				
2	42	28	45	28
3	47	25	48	26
4	42	26	39	23
5	123	113	130	106
*6*	*39*	*23*	*39*	*27*
*7*	*53*	*27*		
*8*	*70*	*25*	*67*	*25*
*9*	*45*	*30*	*46*	*30*
*10*	*35*	*15*	*41*	*27*
*11*	*82*	*26*	*100*	*52*
*12*	*56*	*23*	*79*	*40*

Cohort 2 is shown in italics. No data was available for patient number 1. The left eye of patient number 7 was not included in the analysis because of a retinal detachment

### Cohort 2

**Fig. 1:**
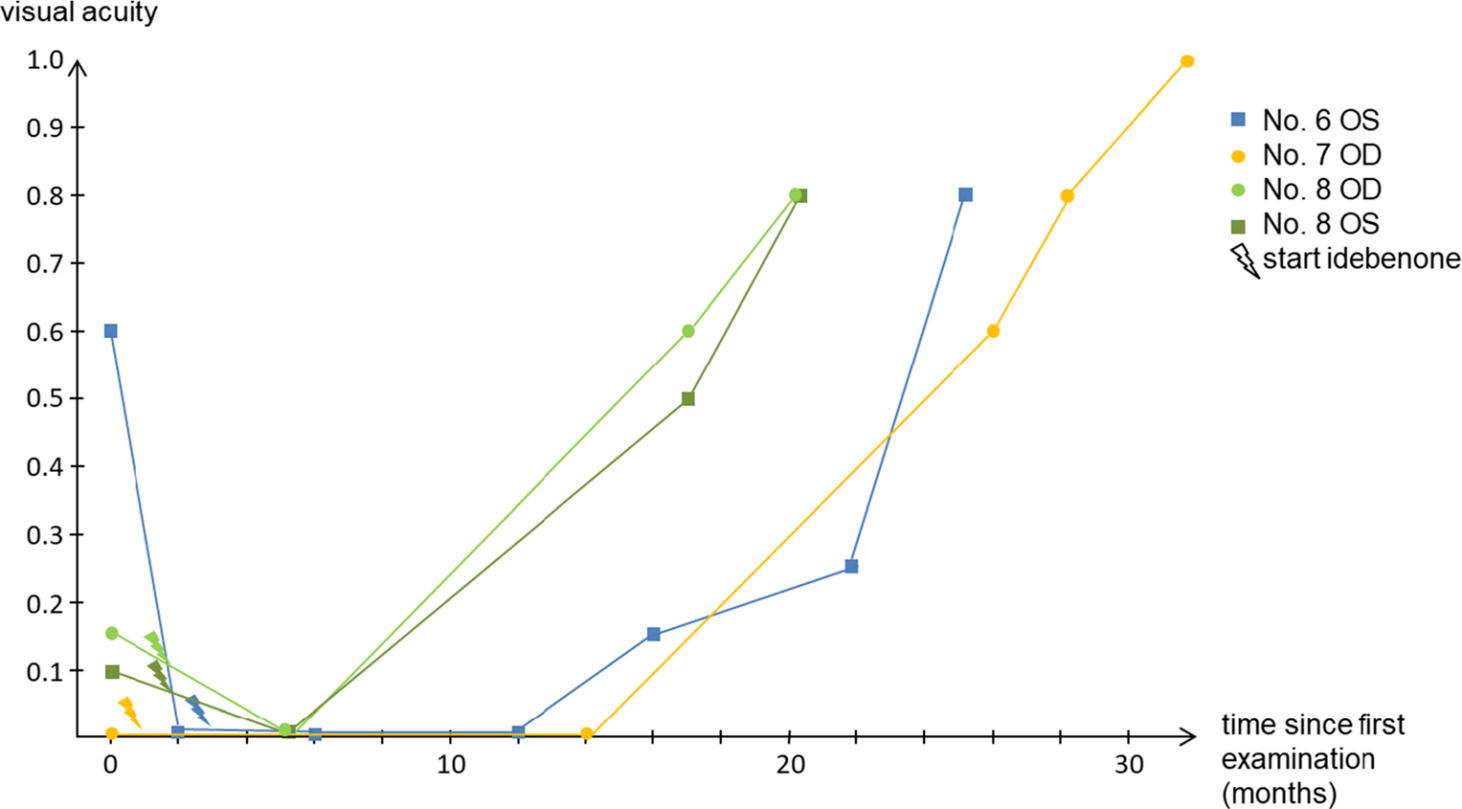
Timeline of visual acuity of responders (cohort 2). OD of no. 6 is not shown due to lack of improvement, OS of No. 7 due to retinal detachment

In cohort 2, four patients carried the m.11778G>A mutation. MT-ND1-3733, MT-ND1-3635, and m.3460G>A occurred once each. Primary VA ranged between 0.03 and 0.63 (median 0.03) in the right eye and 0 and 0.63 in the left (median 0.06). Two patients suffered from a deterioration of their better eye (VA 0.6 to 0.05 and 0.63 to 0.04) 2 and 1 months after onset of first symptoms, respectively. VA improved in 3 patients: A beginning improvement of the left eye of patient number 6 was observed 13 months after start of idebenone treatment (0.05 to 0.8); in the only remaining right eye (preexisting retinal detachment in the other eye) of patient number 7, a beginning improvement was observed 25 months after start of idebenone treatment (0.03 to 1.0) and an improvement of both eyes of patient number 8 was first observed 15 months after beginning of idebenone treatment (right eye 0.16 to 0.8, left eye 0.1 to 0.8). All patients showing a recovery of VA carried the m.11778G>A mutation. The exact course of the responder’s visual acuity is shown in Fig. 1.

Final VA of the non-responding patients differed after median 22 months (range 12–35 months) between 0.0052 and 0.01 for the right eye (median 0.0076) and 0 and 0.5 (median 0.03) for the left. Homoplasmy was present in 5 patients; heteroplasmy in 2 patients. Homoplasmy was true for all patients with improvement of VA.

Table 3 shows the global and temporal RNFL thicknesses of all patients. The median global RNFL of cohort 2 was 53 μm (35–100); the temporal RNFL 27 μm (15–52).

### Cohorts 1 and 2

The difference in RNFL thickness between cohorts 1 and 2 was not significant. The median global and temporal RNFL thicknesses of only the improved eyes were 60 μm (39–70) and 26 μm (25–27), respectively, while all unimproved eyes of both cohorts had thicknesses of 46 μm (35–130) and 27 μm (15–113), respectively. Due to the small number of improved eyes, a statistical evaluation of this aspect is not possible.

For both cohorts, we found no influence of age, sex, or homo-/heteroplasmy on the development of visual acuity.

## Discussion

LHON is a disease not yet fully understood. Neither is it known why the time of onset of the disease varies between childhood and old age, nor why in certain cases spontaneous remissions occur. What is known is that among the three primary LHON mutations, m.14484T>C has the best prognosis of spontaneous visual improvement with 37–71%. For the USA and Europe, spontaneous remissions for m.3460G>A and m.11778G>A have been reported in 4% [3, 4], whereby up to 17% of Japanese patients may show such a favorable course [5]. Although our case numbers are too small for statistical evaluation, a spontaneous remission of three out of four m.11778G>A patients with a final visual acuity of 0.8–1.0 of the affected eyes seems unlikely. Since these patients all belong to cohort 2 treated with idebenone, a connection is conceivable.

In a placebo-controlled, randomized phase III study Klopstock et al. reported that idebenone treatment (900 mg daily) may have a positive effect on LHON [8]. But the effect was small and limited to patients with discordant VA which means a short duration of illness. It is possible that the duration of the study of 22 weeks was too short to obtain clearer results. Nevertheless, an international consensus statement recommended that idebenone should be the standard therapy for genetically confirmed LHON in the first year after disease onset [9].

Moon et al. found in their cohort that younger age at onset and a less severe reduction of VA at the nadir were associated with a higher probability of spontaneous visual recovery [10]. These characteristics were not valid in our small patient cohort. Another publication showing a possible improvement under idebenone treatment was contributed by Pemp et al. [11]: Idebenone treatment over 1 year led to an increase in VA by an average of − 0.2 logMAR in patients who had been symptomatic for 5 years or longer. It remains unclear whether a longer duration of the study would have led to further improvement or whether the long time since the onset of the disease prevented stronger improvement.

In both our cohorts, some patients showed a decrease in VA over time. This seems typical for the normal course of LHON because only patients with newly emerged disease have been included in the study and at the time of the initial examination the worst visual acuity had not been developed in all eyes.

Patient no. 6 showed recovery in only one eye. The question arises why the systemic treatment has not led to an improvement in both eyes. Analysis of the OCT data shows that this patient has a lower RNFL thickness of the temporal quadrant (23 μm) compared to the other patients. A possible explanation would be that structural damage of a certain degree prevents a functional improvement in the sense of a point of no return.

The RNFL analysis showed a better global thickness and a slightly lower temporal thickness for the improved eyes compared to all non-improved eyes. The reason for this is not known. Perhaps the process of improvement prevents further structural damage to the optic nerve.

Recovery of VA occurred in 3 out of 7 LHON patients. As Fig. 1 shows, VA improvement was observed 13–25 months after beginning of idebenone treatment. Even if an earlier start of improvement cannot be excluded due to the intervals between examinations, the VA’s timeline indicates that idebenone should be given 18 months in order to be able to determine the beginning of an improvement with certainty. The trajectory of the VA graph shows an improvement until the end of the observation period; therefore, it seems possible that even after the end of the observation period an improvement occurred. However, such data are not yet available.

All improved patients carried the m.11778G>A mutation, which in itself has the worst spontaneous prognosis of the three main mutations. The high fraction of improving patients among those who usually have a poor prognosis could indicate a significant therapeutic effect. The time course of improvement suggests that beyond the international consensus statement [9] idebenone should be given 1.5 years in newly diagnosed LHON cases. Further studies on this subject are desirable to better understand how idebenone can influence the course of the disease.
